# Supplementary material for: MetaRibo-Seq measures translation in microbiomes
Source: Nat Commun. 2020 Jun 29;11:3268. doi: 10.1038/s41467-020-17081-z (PMC7324362; doi:10.1038/s41467-020-17081-z)
Supplement: Supplementary file 10 — Supplementary Data 7 [file 41467_2020_17081_MOESM10_ESM.zip › File2/Confidence_High_Taxonomy/224292_out.krona.html]

Javascript must be enabled to view this page.

members
magnitude
magnitudeUnassigned
count
unassigned
taxon
rank

224292\_out

6

2
superkingdom
6

1239
phylum
6

186801
6
class

order
6
186802

family
3
186803

genus
2
28050

2

SRS104084\_contig\_number\_2239SRS149879\_contig\_number\_21874
species
446043

1898203
species
1

SRS098644\_contig\_number\_36077

1897043
3
species

SRS015431\_contig\_number\_54333SRS019068\_contig\_number\_119160SRS064276\_contig\_number\_20291
